# Diffuse skeletal [^18^F]-FDG uptake from renal osteodystrophy with parathyroid hyperplasia in an ESRD patient: A case report

**DOI:** 10.22038/aojnmb.2026.89787.1654

**Published:** 2026

**Authors:** Fatemeh Saboktakin, Saeed Farzanefar, Farahnaz Aghahosseini, Nasim Vahidfar, Niloofar tabatabaeian

**Affiliations:** Department of Nuclear Medicine, Vali-Asr Hospital, Tehran University of Medical Sciences, Tehran, Iran

**Keywords:** ESRD, [^18^F]-FDG PET/CT, Renal osteodystrophy Hyperparathyroidism A B S T R A C T

## Abstract

Secondary and tertiary hyperparathyroidism (HPT) are common complications in patients with end-stage renal disease (ESRD) and may result in renal osteodystrophy. While [^18^F]-FDG PET/CT is primarily used to evaluate malignancy, it may occasionally reveal metabolic or endocrine abnormalities. A 45-year-old male with ESRD on hemodialysis presented with generalized bone pain and radiologic suspicion of malignancy. [^18^F]-FDG PET/CT demonstrated diffusely increased skeletal uptake and multifocal lytic bone lesions, consistent with metabolic bone disease secondary to renal osteodystrophy. Incidental findings of soft tissue nodules in both tracheoesophageal grooves suggested parathyroid pathology. Subsequent [^99m^Tc]-Tc-sestamibi SPECT/CT confirmed parathyroid adenomas/ hyperplasia. Laboratory tests revealed elevated calcium (10.8 mg/dL), phosphorus (7.2 mg/dL), and markedly increased parathyroid hormone (3120 pg/mL) levels.This case highlights that diffuse skeletal [^18^F]-FDG uptake in ESRD may mimic malignancy but actually represent metabolic bone disease due to secondary or tertiary HPT. The [^18^F]-FDG PET/CT findings of parathyroid lesions were incidental, while [^99m^Tc]-Tc-sestamibi SPECT/CT accurately localized the hyperfunctioning glands. FDG PET/CT can incidentally demonstrate metabolic bone disease and parathyroid-[^18^F] abnormalities in ESRD, but [^99m^Tc]-Tc-sestamibi SPECT/CT remains the preferred modality for parathyroid localization. Awareness of this potential finding may prevent misinterpretation and guide appropriate management.

## Introduction

 Hyperparathyroidism (HPT) results from dysregulated calcium and phosphorus metabolism due to excessive PTH secretion (1). Among primary, secondary, and tertiary HPTs, secondary HPT arises from conditions such as chronic renal failure, posthemodialysis, kidney transplantation, and bariatric surgery. It leads to increased PTH secretion in response to low serum calcium levels (2). Tertiary HPT occurs as a compensatory response to prolonged overexpression of PTH, often resulting in parathyroid nodules (2). HPT can cause various dysfunctions including, bone diseases (pathological fractures or skeletal deformities), cardiovascular diseases, and kidney stones and may also induce end-stage parathyroid cancer (3-6). Chronic renal failure, including renal osteodystrophy, can result in a spectrum of diseases affecting the bone in patients with long-standing end-stage renal disease (7). In most cases this dysfunctions are often indicated by elevated bone turnover thereupon chronic hypocalcemia (8). 

 [^18^F]-FDG positron emission tomography/ computed tomography (PET/CT) provides a valuable assessment of both metabolic and morphologic abnormalities. The therapeutic procedures for patients undergoing such imaging will be discussed below.

## Case report

 A 45-year-old male with ESRD on maintenance hemodialysis for 8 years presented with progressive generalized bone pain. Two years earlier, he had undergone spinal surgery at T12–L1 for vertebral collapse related to renal osteodystrophy. Despite medical therapy including phosphate binders, calcimimetics, and vitamin D analogs, his symptoms worsened. Laboratory tests revealed persistent hypercalcemia (10.8 mg/dL), hyperphosphatemia (7.2 mg/dL), and markedly elevated parathyroid hormone (PTH) levels (3120 pg/mL). Given the long dialysis duration and hypercalcemia, tertiary hyperpara-thyroidism was diagnosed, consistent with KDIGO 2017 CKD–MBD criteria. A [^99m^Tc]-Tc-sestamibi SPECT/CT scan demonstrated focal uptake in the left inferior parathyroid gland, compatible with a hyperfunctioning adenoma. The patient underwent subtotal para- thyroidectomy (removal of three and a half glands) postoperatively, his laboratory values improved significantly:

PTH: 3120 → 185 pg/mL 

Calcium: 10.8 → 9.4 mg/dL 

Phosphorus: 7.2 → 4.3 mg/dL 

 The patient experienced marked clinical improvement with resolution of bone pain and recovery of mobility. A subsequent [^18^F]-FDG PET/CT -initially performed to exclude malignancy-showed diffusely increased skeletal uptake with multifocal lytic lesions characteristic of osteitis fibrosa cystica, confirming the high bone turnover state secondary to hyperpara-thyroidism. [^18^F]-FDG PET/CT thus defined the skeletal metabolic pattern but did not localize the parathyroid adenoma, which was clearly demonstrated by [^99m^Tc]-Tc-sestamibi SPECT/CT. Follow-up imaging confirmed surgical success and metabolic remission.

**Figure 1:**
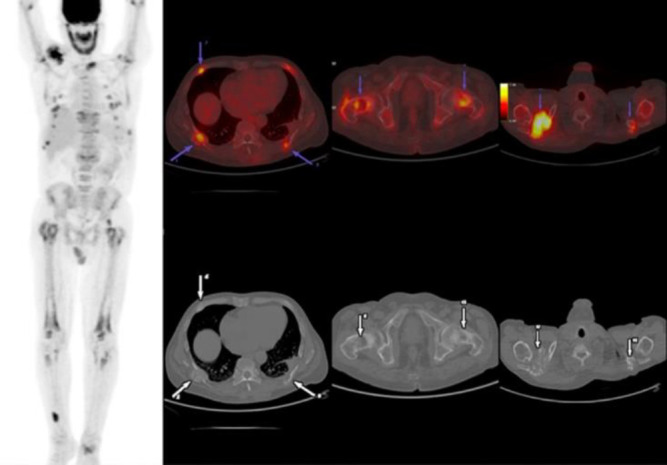
Whole-body anterior maximum intensity projection (MIP) images from [^18^F]-FDG PET/CT demonstrated diffusely increased uptake in the axial and appendicular skeleton, with absent of radiotracer excretion in the kidneys and urinary bladder. Axial CT and fused PET/CT images showed multifocal lytic lesions with increased uptake in clavicles, right scapula, bilateral ribs, multiple vertebrae, pelvic bones and right tibia indicating multifocal osteitis fibrosa cystica (SUV_max _up to 9.9, measuring up to 8×5×7cm in right scapula)

**Figure 2:**
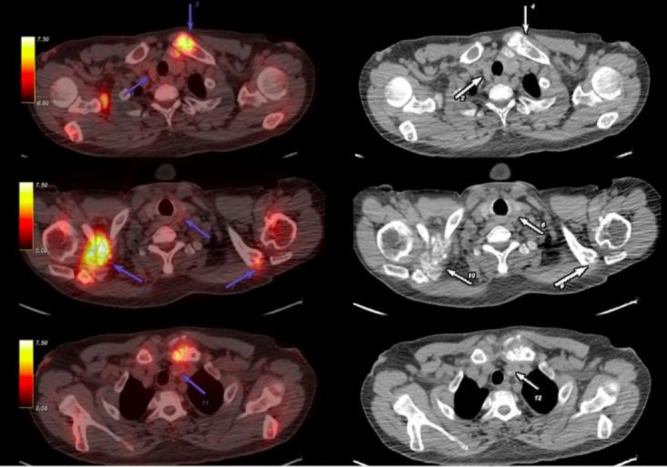
Axial fused PET/CT slices showed three soft tissue density nodules in both tracheoesophageal grooves (two of them located posterior to upper and lower left thyroid lobes and one posterior to right lower thyroid lobe) without significant [^18^F]-FDG uptake (SUV_max_ up to 1.8) measuring up to 20×25mm in left side and 20×10mm in right side, consistent with parathyroid adenomas/hyperplasia. Also multiple bone lesions are noted which are most likely lytic in bilateral clavicles (SUV_max_ up to 6.9 in left clavicle) and bilateral scapulae, more prominent in right side (SUV_max_ up to 9.9) as shown in images

## Discussion

 HPT is a frequent complication of ESRD due to chronic disturbances in calcium–phosphate metabolism (9, 10). According to the KDIGO 2017 CKD–MBD guidelines, long-standing secondary HPT in dialysis patients can progress to tertiary HPT characterized by autonomous parathyroid hyperfunction and hypercalcemia (9, 11). In this patient, prolonged dialysis, persistent hypercalcemia, and markedly elevated PTH (>3000 pg/mL) established the diagnosis of tertiary HPT. The [^18^F]-FDG PET/CT demonstrated diffuse skeletal uptake with multifocal lytic lesions, a pattern mimicking malignant or infiltrative bone disease (12). However, several factors excluded metastatic disease, multiple myeloma, inflammatory skeletal disorders, and bone marrow hyperplasia (13). The uptake pattern was uniformly diffuse and symmetric across axial and appendicular skeleton, without discrete focal hypermetabolic lesions typical of metastases. There was no evidence of marrow plasmacytosis or lytic foci with soft-tissue components suggestive of myeloma on CT correlation. Inflammatory or infectious causes were ruled out given the absence of systemic symptoms, low CRP, and no localized osteitis or abscess. Bone marrow hyperplasia, while showing diffuse uptake, typically occurs in anemia or hematologic stimulation - neither present in this patient. Therefore, the diffuse “superscan-like” appearance was attributed to high bone turnover and osteitis fibrosa cystica secondary to renal osteodystrophy, consistent with prior reports describing [^18^F]-FDG superscans in ESRD (14). Importantly, the [^18^F]-FDG PET/CT defined the skeletal metabolic activity but did not localize the parathyroid adenoma, as [^18^F]-FDG uptake in parathyroid lesions is variable. In contrast, the [^99m^Tc]-Tc-sestamibi SPECT/CT scan clearly identified a hyperfunctioning gland, in line with the EANM 2009 Parathyroid Guidelines recommending MIBI scintigraphy as the standard imaging technique for preoperative localization (15). 

 This case illustrates that while [^18^F]-FDG PET/CT may incidentally reveal the metabolic sequelae of tertiary HPT, it should not replace dedicated parathyroid imaging. Recognizing this metabolic “superscan” pattern is crucial to avoid misdiagnosing malignancy and to guide appropriate endocrine and surgical management. 

## Conclusion

 In ESRD patients, diffuse skeletal [^18^F]-FDG uptake can mimic metastatic disease but often represents osteitis fibrosa cystica secondary to secondary or tertiary hyperparathyroidism. In this case, [^18^F]-FDG PET/CT characterized the metabolic skeletal pattern, while [^99m^Tc]-Tc-sestamibi SPECT/CT accurately localized the parathyroid adenoma. Subtotal parathyroidectomy achieved biochemical normalization and clinical recovery FDG PET/CT may incidentally reveal metabolic bone disease in ESRD, but [^99m^Tc]-Tc-sestamibi SPECT/CT remains the modality of choice for parathyroid localization. 
